# Association between obesity and lung function in South African adolescents of African Ancestry

**DOI:** 10.1186/s12887-022-03164-x

**Published:** 2022-02-28

**Authors:** Godwill Azeh Engwa, Chungag Anye, Benedicta Ngwenchi Nkeh-Chungag

**Affiliations:** 1 Department of Biological and Environmental Sciences, Faculty of Natural Sciences, Walter Sisulu University PBX1, Mthatha, 5117 South Africa; 2 Dayenuel Consulting, Postnet Suites 092, Mthatha, 5099 South Africa

**Keywords:** Obesity, Lung function, Airway obstruction, Adolescent

## Abstract

**Background:**

There is a growing body of literature on the increasing prevalence of obesity in adolescents of Sub-Saharan African ancestry. However, limited data is available on the impact of obesity on pulmonary function. This study assessed the relationship between obesity and lung function in South African adolescents of African ancestry.

**Methods:**

This was a cross-sectional study involving 10–14 year old adolescents recruited from middle schools of the Eastern Cape Province of South Africa. Anthropometric measurements were performed. Body mass index (BMI) was converted to percentiles for age and sex and used to classified obesity. Spirometry was performed to assess lung function. Chi-square test of association and binary regression analysis were used to assess the relationship between obesity and airway obstruction. Adjusted linear regression was used to determine the relationship between obesity and lung function parameters.

**Results:**

A total of 540 adolescents were recruited for the study among which 77 (14.3%) were obese. Lung function parameters: forced vital capacity (FVC) and forced expiratory volume in 1 s (FEV_1_) were higher (*p* < 0.001) in obese than in non-obese adolescents while peak expiratory flow (PEF) % and FEV_1_/FVC ratio were lower (*p* < 0.05) in obese than non-obese adolescents. Obesity was associated (χ2 = 9.614; *p* < 0.01) with airway obstruction and obese adolescents were over 1.5 times more likely to have pulmonary obstruction (OR: 1.57; *p* < 0.05) than their non-obese counterparts. Anthropometric measures were positively associated (*p* < 0.05) with FVC, FEV1, PEF and/or FEV_25-75_ but negatively associated with FEV_1_/FVC ratio.

**Conclusions:**

Obesity was associated with airway obstruction in South Africa adolescents of African ancestry.

## Background

Obesity is a global epidemic known to increase the risk of cardiovascular diseases (CVDs), hypertension, type 2 diabetes, and certain forms of cancer [1]. The prevalence of obesity and overweight is continuously on the rise and almost doubling since 1980 with 1.9 billion obese and 609 million overweight adults in 2015. This translates to over a third (39%) of the world’s population affected [2]. The world has equally observed a drastic rise in the prevalence of obesity in children. Recent reports suggest over 40 million children below 5 years of age and more than 330 million children and adolescents aged 5–19 years were either obese or overweight in 2016 [3, 4]. Global analyses show that a vast majority of obese children aged between 5 and 19 years are within the African region with the largest population in the Southern African region [5].

Obesity is a chronic disease characterized by abnormal or excessive body fat accumulation that may impair health. It is commonly assessed by measuring body mass index (BMI), which highly correlates with body fat and is therefore a useful measure in routine clinical assessment and epidemiological studies [6]. BMI percentile (pBMI) which is relative to gender and age is the recommended measure to assess obesity in children of all ages [7].

Obesity is known to be associated with respiratory diseases such as asthma and chronic obstructive pulmonary disease (COPD) [8, 9]. Excess adipose tissue as a result of obesity exerts a mechanical effect on the lung and affects the respiratory well-being of affected persons since it increases the consumption of oxygen and production of carbon dioxide, while at the same time it increases the mechanical workload for breathing and stiffens the respiratory system [10]. Excess adipose tissue in the chest wall and abdominal cavity compresses the lungs, diaphragm, and thoracic cage, thus reducing the diaphragm’s capacity to move downward limiting inflation of the lung [11]. Also, adipose tissue in the thoracic region reduces the volume of the chest cavity and limits movements in the chest wall. A decrease in chest and lung wall compliance coupled with a decrease in diaphragm displacement, and a corresponding increase in elastic recoil results in an overall decrease in lung volume which overloads the inspiratory muscles of the chest [12]. Thus, increase in fat in the thoracic region can affect respiratory function such as decrease in the strength and endurance of the respiratory muscle, alterations in respiratory mechanics, lower control of breathing, decrease in pulmonary gas exchange and airway obstruction [13, 14]. These pulmonary changes are exacerbated by increases in the BMI [15]. The common physiological abnormalities due to obstructive mechanics of the lungs are manifested as changes in the force expiratory volume (FEV) and forced vital capacity (FVC) of the lungs which are commonly assessed by spirometry, a primary screening tool for lung function [16].

Reports in South Africa have shown a high prevalence of obesity in adolescents [17, 18] with some studies by our group in children of African ancestry showing between 30 and 40% prevalence of obesity/overweight [19–21]. Although obesity is highly prevalent in South African adolescents, little is known about its impact on pulmonary function. Several studies in children have shown an inverse association between obesity and respiratory function impairment [22, 23]. while others failed to show any relationship [24]. Therefore, this study aimed to assess the relationship between obesity and pulmonary function in South African adolescents.

## Methods

### Ethics consideration

This study was conducted in accordance with the Helsinki Declaration guidelines (2008 revised version). Ethical approval was obtained from the Walter Sisulu University Health Sciences Ethics Committee (Ref No: 112/2018). Study objectives and procedures were explained after which written informed consent was obtained from parents/legal guardians of participants as well as the relevant school authorities before enrolment into the study. Participation was voluntary and participants could freely withdraw from the study at any point. The study was in accordance with the National Data Protection Act and adhered to the standards of reporting clinical data wherein participants were assigned specific codes, and their data/samples were stored anonymously. No important changes were made in the study methods after commencement.

### Study design

This was a cross-sectional study that recruited adolescents aged 10–14 years from some selected middle schools of the Eastern Cape Province of South Africa.

### Inclusion/exclusion criteria

Adolescents aged 10–14 years (males or females) who were free from any CVDs, renal, or pulmonary diseases were enrolled into the study. Physically challenged and sick individuals with fever as well as individuals on weight loss therapy or blood pressure lowering medication were excluded from the study.

### Anthropometric measurements

Anthropometric measurements were performed in accordance with the guidelines of the International Standards for Anthropometric Assessments [25] and as previously reported by us [26]. Participants were requested to stand upright with feet together and their waist circumference (WC) and hip circumference (HC) were measured with a measuring tape in centimeters (cm). The WC and HC were used to calculate waist to hip ratio (WHR). Participants were requested to take off their shoes and socks and to step on the wall-mounted stadiometer (Electronic Body Scale TCS-200-RT) platform, close to the stadiometer rod. A movable bar on the stadiometer was lowered to just touch their head and their height was measured to the nearest cm. Weight, total fat mass (TFM) and total muscle mass (TMM) were measured using an Omron body composition monitor (BR511). Participants personal data including age, height, and sex were entered into the Omron monitor to calculate the body mass index (BMI) expressed as weight/height^2^ (kg/m^2^). The BMI was converted to percentiles (pBMI) for sex and age according to the CDC criteria and a pBMI ≥ 95^th^ percentile was categorised as obesity [27]. The weight and height were used to calculated the weight to height ratio (WHtR).

### Blood pressure measurements

Blood pressure (BP) was measured using appropriate arm cuff fitted on the left arm of participants using the Omron (Hem 7120) automated blood pressure machine after resting for ten minutes in a seated position. Measurements were done in triplicates at three-minute intervals. The mean of three readings of the diastolic blood pressure (DBP), systolic blood pressure (SBP) and heart rate (HR) were calculated.

### Pulmonary function test

**Fig. 1:**
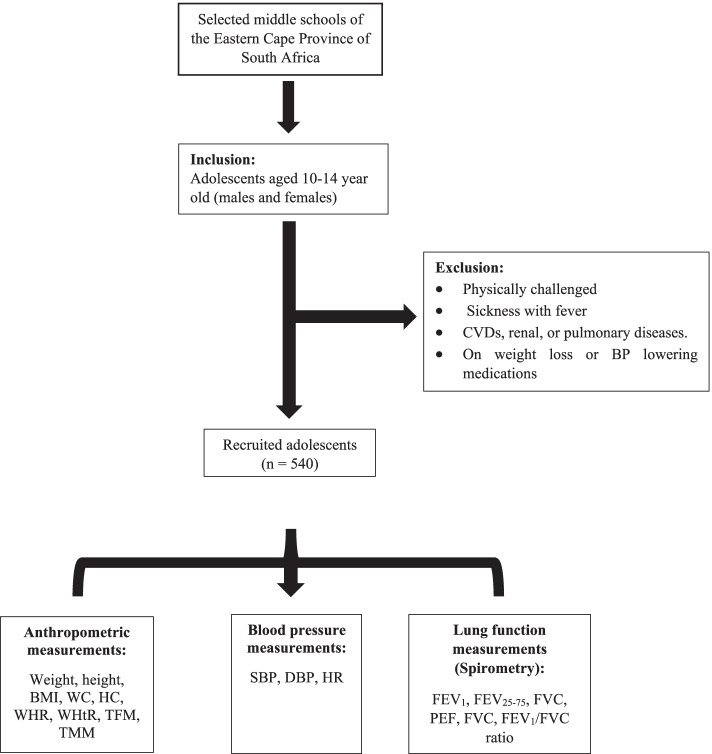
Study design flow chart

Lung function test was performed in accordance with the ATS/ERS guidelines [28] using a Contec hand-held spirometer SP10 (Contec Medical Systems Co., Ltd, Qinhuangdao, China). Prior to the testing, participants’ age, weight and height were computed into the device. After participants sat in an upright position and rested for at least 5 min, a nose clip was worn and the test was conducted as per device protocol. The parameters that were assessed by the device included the forced expiratory volume in 1 s (FEV_1_), forced vital capacity (FVC), the peak expiratory flow (PEF), forced expiratory volume 25–75% interquartile (FEV_25-75_) and ratio of FEV_1_ to FVC (FEV_1_/FVC) calculated. After measurement, the best three acceptable readings were recorded and the mean calculated. The device was disinfected after every participant and the mouthpiece replaced. The study design flow chart is summarised in Fig. 1.

### Statistical analysis

Data were analysed using Statistical Package for Social Sciences (SPSS) Version 20 (IBM. Inc, 1 New Orchard Road Armonk, New York, USA). Data were summarised in tables as mean ± standard deviation. Independent sample t-test was used to compare mean differences of quantitative variables between males and females as well as between obese and non-obese participants. Chi-square test of association and binary regression analysis were used to assess the relationship between obesity and airway obstruction. Linear regression was employed to assess the association between obesity and lung function parameters after adjusting for age and sex. Differences with a *p*-value ≤ 0.05 were considered significant.

## Results

### Characteristics of study population

Five hundred and forty (540) adolescents were recruited for the study: 290 (53.7%) were females and 240 (44.3%) were males. Females were heavier than males (*p* < 0.001) and all the anthropometric measurements and ratios (WC, HC, WHtR, BMI, pBMI, TFM and TMM) except for WHR were higher in females than in males *(p* < 0.01). Among the 540 participants, 77 (14.3%) were obese: 44 (8.1%) females and 33 (6.2) males. Blood pressure parameters (SBP, DBP and HR) were different between males and females (*p* < 0.005). On the other hand, only the PEF% for lung function was different between males and females (*p* < 0.05) (Table 1).

**Table 1:** Characteristics of study population

	**Female**	**Male**	***p*-value**
Weight	46.99 ± 17.33	41.94 ± 15.32	< 0.001
Height	148.11 ± 16.47	145.62 ± 16.43	0.081
WC	68.96 ± 12.42	65.18 ± 10.62	< 0.001
HC	85.03 ± 14.11	79.55 ± 12.27	< 0.001
WHR	0.81 ± 0.06	0.82 ± 0.09	0.119
WHtR	0.46 ± 0.07	0.44 ± 0.07	0.001
BMI	20.33 ± 5.22	18.96 ± 4.13	0.001
pBMI	57.79 ± 31.76	49.74 ± 32.61	0.004
TFM	23.97 ± 10.02	19.11 ± 8.96	< 0.001
TMM	32.64 ± 3.01	34.95 ± 3.59	< 0.001
SBP	112.63 ± 10.28	110.11 ± 10.36	0.005
DBP	73.14 ± 7.64	70.57 ± 7.28	< 0.001
HR	90.04 ± 11.84	84.26 ± 12.54	< 0.001
FVC	2.01 ± 0.43	2.06 ± 0.52	0.207
FVC%	76.95 ± 11.96	75.02 ± 12.23	0.066
FEV_1_	1.79 ± 0.35	1.81 ± 0.43	0.616
FEV_1_%	74.35 ± 11.43	76.52 ± 11.67	0.030
PEF	4.25 ± 1.31	4.09 ± 1.26	0.152
PEF%	93.30 ± 23.45	84.22 ± 21.95	< 0.001
FEV_1_/FVC	0.90 ± 0.08	0.89 ± 0.08	0.130
FEV_25-75_	2.48 ± 0.71	2.34 ± 0.69	0.016

*Legend: HC* Hip circumference, *WC* Waist circumference, *WHR* Waist to hip ratio, *TMM* Total muscle mass, *TFM* Total fat mass, *BMI* Body mass index, *pBMI* Body mass index percentile, *WHtR* Weight to height ratio, *SBP* Systolic blood pressure, *DBP* Diastolic blood pressure, *HR* Heart rate, *FEV*_*25-75*_ Forced expiratory volume 25–75% Interquartile, *FEV*_*1*_ Forced expiratory volume in 1 s, *FVC* Forced vital capacity, *PEF* Peak expiratory flow

### Obesity and lung function

Lung function parameters (FVC and FEV_1_) were higher (*p* < 0.001) in obese than in non-obese adolescents while PEF% and FEV_1_/FVC ratio were lower (*p* < 0.05) in obese than non-obese adolescents (Table 2).

**Table 2:** Lung function parameters between obese and non-obese adolescents

	**Non-obese**	**Obese**	***p*-value**
FVC	2.00 ± 0.45	2.24 ± 0.56	< 0.001
FVC%	75.77 ± 12.00	78.79 ± 12.73	0.176
FEV_1_	1.78 ± 0.37	1.95 ± 0.47	< 0.001
FEV_1_%	75.21 ± 11.34	76.21 ± 13.03	0.486
PEF	4.19 ± 1.29	4.09 ± 1.28	0.534
PEF%	90.14 ± 23.35	82.91 ± 21.35	0.011
FEV_1_/FVC	0.90 ± 0.08	0.88 ± 0.09	0.050
FEV_25-75_	2.40 ± 0.70	2.47 ± 0.75	0.431

*Legend: FEV*_*25-75*_ Forced expiratory volume 25–75% interquartile, *FEV*_*1*_ Forced expiratory volume in 1 s, *FVC* Forced vital capacity, *PEF* Peak expiratory flow

### Obesity and airway obstruction

As shown in Table 3, the proportion of obese adolescents with airway obstruction (7.8%) was higher than the prevalence in non-obese adolescents (1.7%) translating to an association between obesity and airway obstruction (χ2 = 9.614; *p* < 0.01). Obese adolescents were over 1.5 times more likely to have pulmonary airway obstruction (OR: 1.57; *p* < 0.05) than their non-obese counterparts.

**Table 3:** Relationship between obesity and airway obstruction

	**Non-obese**	**Obese**	**Total**	**χ2**	***p*-value**
AO	8 (1.7)	6 (7.8)	14 (2.6)	9.614	0.008
Normal	455 (98.3)	71 (92.2)	526 (97.4)	**OR**	***p*-value**
Total	463	77	540	1.57	0.05

*AO* Airway obstruction, *χ2* Chi-square test, *OR* Odd ratio

### Relationship between lung function and anthropometric measures

The relationship between anthropometric indices and lung function parameters showed increased HC, WC, WHtR, pBMI, TFM and TMM to associate (*p* < 0.05) positively with FVC. Increasing HC, WC, pBMI, TFM and TMM were associated (*p* < 0.05) with an increase in FEV_1_. Increased PEF was associated (*p* < 0.05) with an increase in HC, WC, pBMI, and TMM. Increase in WC, pBMI and TMM were associated (*p* < 0.05) with an increase in FEV_25-75_. Moreover, increased HC, WC, pBMI and TMM were associated (*p* < 0.05) with reduced FEV_1_/FVC ratio (Table 4).

**Table 4:** Predictors of lung function parameters in children

Regression coefficient	HC	WC	WHR	WHtR	pBMI	TFM	TMM
FVC	0.013***	0.012***	-0.236	0.703*	0.004***	0.004*	0.03***
FEV_1_	0.009***	0.009***	-0.284	0.382	0.003**	0.004*	0.023***
PEF	0.015**	0.017***	-1.036	-0.319	0.005**	0.003	0.061***
FEV_1_/FVC	-0.001**	0.001**	0.025	0.088	-0.002*	0.001	-0.001
FEV_25-75_	0.008	0.009***	-0.733	0.118	0.003**	0.005	0.023**

*Legend:* *significant difference at *p* < 0.05; **significant difference at *p* < 0.01; ***significant difference at *p* < 0.001. *HC* Hip circumference, *WC* Waist circumference, *WHR* Waist to hip ratio, *TMM* Total muscle mass, *TFM* Total fat mass, *pBMI* Body mass index percentile, *WHtR* Weight to height ratio*, FEV*_*25-75*_ Forced expiratory volume 25–75% interquartile, *FEV*_*1*_ Forced expiratory volume in 1 s, *FVC* Forced vital capacity, *PEF* Peak expiratory flow

## Discussion

In this study we assessed the cross-sectional association between obesity and lung function. The main finding showed that obesity was associated with a decrease in FEV_1_/FVC ratio (airway obstruction) and increase in FVC and FEV_1_ in 10–14 year old adolescents. This finding corroborates with some previous spirometry studies in children and adolescent [29–31].

Obesity has been reported to affect lung function and promote the development of respiratory diseases [10]. In obesity, excess fat is deposited in the mediastinum, chest, and abdominal cavity which affects the mechanical properties of the chest and lung wall thus, reduce the compliance of the chest and lung wall, and the entire respiratory system [32]. This may result in airway closure and narrowing with increased resistance in the respiratory system due to the restricted outward movement of the chest wall and downward movement of the diaphragm. This results in restricted expansion of the lung. On the other hand, airway obstruction which is commonly characterised by an FEV_1_/FVC less than 70% [33] is strongly associated with obesity in children [34]. Most studies in adults have shown that obese individuals (with or without asthma) have a reduced FEV_1_ and FVC but present a normal FEV_1_/FVC ratio which is suggestive of a restrictive ventilatory deficit [15, 35]. However, although the relationship between obesity and pulmonary function in children and adolescent remains inconclusive [16] several studies have shown obesity to be associated with reduced FEV_1_/FVC (an obstructive deficit) [36, 37]. Findings in this study showed that FEV_1_/FVC was significantly lower in obese adolescents. Moreover, a negative association was observed between obesity parameters (WC, HC and pBMI) and FEV_1_/FVC ratio in these adolescents. Also, there was an association between obesity and airway obstruction as obese adolescents were over 1.5 times more likely to have airway obstruction. These findings suggest that obesity was associated with airway obstruction in adolescents. Previous studies have equally reported airway obstruction in obese children and adolescents [38, 39].

Although obese individuals have been reported to have a reduction in FEV_1_ and FVC [15, 35], studies in children have reported an increase in FEV_1_ and FVC and a reduction in FEV_1_/FVC in obese children [31, 40]. In fact, Lazarus et al. showed that even after adjusting for height, an increased FEV_1_ and FVC, and a lower FEV_1_/FVC ratio was associated with obesity in children [40]. Findings in this study showed FEV_1_ and FVC to be increased in obese adolescents and there was a positive association between obesity parameters (WC, HC, WHtR, BMI, TFM) and lung function parameters (FEV_1_, FEV_25-75_, FCV and PEF) in these adolescents. These findings suggest that childhood obesity is associated with increased FEV_1_ and FVC in this population. This finding is in accordance with some previous studies that have shown increased FVC and FEV_1_ and reduced in FEV_1_/FVC in obese children [31, 41]. This increase in FVC and FEV_1_ in obese adolescents which is unexpected has been suggested by previous studies to be due to airway dysanapsis [42, 43], a condition characterised by disproportionate relationship between airways and lungs. Airway dysanapsis is a condition which results from an incongruence of a faster growth in airway length and lung volume relative to a slower rate of increase in airway caliber [42]. Thus, children with obesity experience an accelerated pace of lung growth resulting in airway dysanapsis evident by a disproportionate increase in FVC compared to FEV_1_. This incongruence may be a natural physiological process which originated early in life. A meta-analysis study of 24 birth cohorts which recruited over 25,000 children by den Dekker and colleagues found that greater weight at birth and weight gain at infancy were associated with higher FVC and FEV_1_ at school age irrespective of the gestational age of the children [44]. Moreover, lower FEV_1_/FVC and FEV_25-75_ were associated with infant weight gain. Thus, these changes observed may have originated early in life following increased weight after birth.

The strength of this study is that it utilised a relatively sufficient sample size to assess the relationship between obesity and lung function in adolescents of African ancestry. Thus, the findings of this study are reliable and dependable. Further, this is one of the first reports on the relationship between obesity and pulmonary function in South African adolescents of African ancestry.

Despite these strengths, this study was limited to spirometry analysis. Other parameters of lung function assessment including total lung capacity (TLC), functional residual capacity (FRC), and expiratory residual volume (ERV) were not assessed due to the lack of whole-body plethysmography [45, 46]. This was a cross-sectional study and thus did not selectively isolate adolescents with airway obstruction or presenting clinical signs and symptoms for dysanapsis to further confirm the impact of obesity on respiratory function. From a public health perspective, the findings of this study have shown associations between obesity and airway obstruction in South African adolescents of African ancestry which is of public health concern and therefore, identifying some modifiable risk factors such as obesity is of importance for the prevention of potential respiratory diseases. Though the sample size was large, the proportion of obese adolescents was relatively small. As such, it will be interesting to have an increased population of obese children to assess the observed finding. Thus, further studies with a larger sample size using longitudinal models will be important to better assess the impact of obesity on lung function indicators including airway obstruction and dysanapsis with associated clinical manifestations.

## Conclusion

This study revealed that obesity was associated with airway obstruction in South African adolescents of African ancestry. This is the first report on the relationship between obesity and lung function in South African children of African ancestry. Being a cross-sectional study, it is therefore necessary for further studies using longitudinal models to ascertain this finding.

## Data Availability

All data generated or analysed during this study are included in this published article.

## Abbreviations

BMI: Body mass index
BP: Blood pressure
DBP: Diastolic blood pressure
COPD: Chronic obstructive pulmonary disease
CVDs: Cardiovascular diseases
ERV: Expiratory residual volume
FEV: Force expiratory volume
FEV_1_: Forced expiratory volume in 1 s
FEV_25-75_: Forced expiratory volume 25–75% interquartile
FRC: Functional residual capacity
FVC: Forced vital capacity
HC: Hip circumference
HR: Heart rate
pBMI: BMI percentile
PEF: Peak expiratory flow
SBP: Systolic blood pressure
SPSS: Statistical Package for Social Sciences
TFM: Total fat mass
TLC: Total lung capacity
TMM: Total muscle mass
WC: Waist circumference
WHR: Waist to hip ratio
WHtR: Weight to height ratio
